# ADHD, Lead, and PCBs: Appropriate Comparison Studies

**DOI:** 10.1289/ehp.1103513

**Published:** 2011-07-01

**Authors:** Jack Brondum

**Affiliations:** Epidemiology and Environmental Health, Hennepin County Department of Human Services and Public Health, Hopkins, Minnesota, E-mail: jack.brondum@co.hennepin.mn.us

In their article “Lead and PCBs as Risk Factors for Attention Deficit/Hyperactivity Disorder” (ADHD), Eubig et al. (2010) offered a large compilation of human and animal research supporting a relationship between these environmental contaminants and ADHD occurrence. Key to understanding such a relationship, however, is research quality, not quantity.

As Eubig et al. (2010) noted, ADHD is highly heritable, a history of ADHD in a parent or sibling being a strong predictor of ADHD occurrence in a child (Faraone and Doyle 2001). A sound study of the disorder and lead or polychlorinated biphenyls (PCBs) would therefore control for family history. The authors listed seven studies of lead exposure and ADHD in their Table 2, but five of the studies had no information on family history so they could not answer the question of a relationship. Another study suffered from likely underascertainment of parental history; even so, it remained significantly (*p* < 0.01) associated with ADHD in case children (Wang et al. 2008). The last study controlled for familial neuropsychiatric disease and reported no significant association of children’s blood lead levels (BLLs) and ADHD, despite its ample cohort size of ≥ 1,700 (Ha et al. 2009).

In their Table 1, Eubig et al. (2010) listed 12 studies of human lead exposure and performance on test functions impaired in ADHD. Only 3 of the studies considered heritability as a possible confounder of this relationship, but none reported an association with performance (Chiodo et al. 2004, 2007; Stewart et al. 2006). This is surprising, given the marked heritability of ADHD, and raises the question of how well individual test functions may control for or serve as surrogates of ADHD diagnosis per se. Also, Stewart et al. (2006) found only a marginal (*p* < 0.047) association with medical record information on postnatal BLL in a potentially biased 60.9% of subjects, and no association (*p* < 0.641) with umbilical cord BLL in 88.6% of subjects.

According to National Health and Nutrition Examination Survey (NHANES) data, the proportion of elevated BLLs (≥ 10 µg/dL) in U.S. children 1–5 years of age dropped from 77.8% in 1976–1980 to 0.9% in 2005–2008 (Centers for Disease Control and Prevention 2005; HealthyPeople.gov 2011). However, the occurrence of ADHD and its diagnostic predecessors has been rising since the 1980s, if not before, offering no support for a positive association of BLL with ADHD (Pastor and Reuben 2008).

The PCB literature Eubig et al. (2010) presented in their Table 4 provided a picture little different from that of lead. PCB exposure is also apparently trending downward (Tee et al. 2003).

The dearth of well-controlled studies leaves open Eubig et al.’s question whether lead or PCBs exert an effect on ADHD occurrence beyond that exerted by heritability.  This question cannot be answered satisfactorily until researchers consistently impose adequate control in their studies and funding agencies consistently require such control in the research they support.
